# Biomechanical parameters of gait among transtibial amputees: a review

**DOI:** 10.1590/S1516-31802009000500010

**Published:** 2010-02-03

**Authors:** Alex Sandra Oliveira de Cerqueira Soares, Edward Yuji Yamaguti, Luis Mochizuki, Alberto Carlos Amadio, Júlio Cerca Serrão

**Affiliations:** I MSc. Assistant professor in the Physiotherapy Department, Universidade de Taubaté (Unitau), Taubaté, São Paulo, Brazil.; II Graduate of Physical Education, School of Physical Education and Sport, Universidade de São Paulo (USP), São Paulo, Brazil.; III PhD. Assistant professor in the School of Arts, Science and Humanities, Universidade de São Paulo (USP), São Paulo, Brazil.; IV PhD. Full professor of the School of Physical Education and Sport, Universidade de São Paulo (USP), São Paulo, Brazil.; V PhD. Associate professor in the School of Physical Education and Sport, Universidade de São Paulo (USP), São Paulo, Brazil.

**Keywords:** Amputation, Biomechanics, Gait, Rehabilitation, Artificial limbs, Amputação, Biomecânica, Marcha, Reabilitação, Membros artificiais

## Abstract

Rehabilitation for lower-limb amputees needs to focus on restoration of daily functions and independent locomotion. As gait is reestablished, reorganization of the motor pattern takes place in order to optimize the functions of the locomotor system. Biomechanics is a field of study that enables understanding of this reorganization. From such knowledge, appropriate strategies for recovering the autonomy of the means of locomotion can be established. Thus, this paper had the aim of reviewing the current status of the biomechanics of locomotion among unilateral transtibial amputees. To achieve this aim, papers written in English or Portuguese and published up to 2005 were selected from the Cochrane Library, PubMed, Scientific Electronic Library Online (SciELO), Literatura Latino-Americana e do Caribe em Ciências da Saúde (Lilacs) and Dedalus databases. In cases of transtibial amputation, the absence of plantar flexors negatively affects locomotion. Increased absorption and energy generation by the muscles that control the hip joint of the amputated leg can be considered to be the main compensatory strategy developed by unilateral transtibial amputees during gait. Factors associated with the characteristics of the amputation, prosthesis and experimental protocol used directly influence the results.

## RESUMO

A reabilitação de amputados de membro inferior deve focar a restauração das funções cotidianas e da locomoção independente. À medida que a marcha é restabelecida, ocorre a reorganização do padrão motor com a finalidade de otimizar as funções do sistema locomotor. A Biomecânica é uma área de estudo que possibilita a compreensão desta reorganização. A partir desse conhecimento, podem-se estabelecer estratégias adequadas para recuperação da autonomia dos meios de locomoção. Assim, este artigo tem por objetivo criar um sumário do atual status do estudo da biomecânica da locomoção de amputados transtibiais unilaterais. Para alcançar tal objetivo foram selecionados artigos nas bases de dados Cochrane Library, PubMed, SciELO (Scientific Electronic Library Online), Lilacs (Literatura Latino-Americana e do Caribe em Ciências da Saúde) and Dedalus escritos em língua inglesa e portuguesa publicados até o ano de 2005. Na amputação transtibial, a ausência dos flexores plantares repercute de forma negativa na locomoção. O aumento da absorção e geração de energia pelos músculos que controlam a articulação do quadril da perna amputada pode ser considerado a principal estratégia compensatória desenvolvida por amputados transtibiais unilaterais durante a marcha. Fatores ligados às características da amputação, prótese e protocolo experimental estudados influenciam diretamente os resultados analisados.

## INTRODUCTION

Reestablishment of independent and functional walking is one of the main focuses of rehabilitation for lower-limb amputees.^1^ In cases of transtibial amputation, which is the level dealt with in the present study, reorganization of the motor pattern is marked by loss of the plantar flexors. This muscle group is responsible for generating about 80% of the mechanical power needed during the gait cycle.^2^ Through description, analysis and synthesis of the knowledge created by biomechanics, the repercussions of this loss can be understood and appropriate rehabilitation strategies for this amputation level can then be established.

Knowledge of locomotion originates from different fields of investigation within biomechanics: kinematics, kinetics and electromyography (EMG). Studies in the field of kinematics have mainly emphasized descriptions of space-time parameters in order to analyze the symmetry between limbs.^3^^,^^4^^,^^5^^,^^6^^,^^7^ In addition, they offer the possibility of evaluating the success of the rehabilitation process.^8^^,^^9^ Studies on kinetics seek to elucidate the mechanisms for control and management of movement,^2^^,^^10^^,^^11^ and to provide ample knowledge of the behavior of the ground reaction force (GRF).^5^^,^^12^^,^^13^^,^^14^^,^^15^ Through electromyography, the main muscle strategies developed can be elucidated.^5^^,^^11^^,^^16^^,^^17^ In all cases, the influence of prosthetic components such as feet,^10^^,^^15^^,^^18^^,^^19^^,^^20^^,^^21^ factors in experimental protocols such as speed^6^^,^^7^^,^^17^^,^^21^ and characteristics of amputation^15^ are noted in relation to movement.

Thus, this article summarizes the findings from each field of biomechanics and then discusses the strategies developed by the locomotor system at this level of amputation.

## METHODS

The Cochrane Library, PubMed, Scientific Electronic Library Online (SciELO), Literatura Latino-Americana e do Caribe em Ciências da Saúde (Lilacs) and Dedalus databases were consulted in order to carry out this review of the literature (Table 1). The key words used were: locomotion, gait, biomechanics and amputation (amputee). English and Portuguese language articles published up to 2008 that satisfied the following inclusion criteria were selected: texts analyzing biomechanical gait parameters for unilateral transtibial amputees and samples consisting of at least three volunteers.

In this paper, the following abbreviations were used: PL for prosthetic leg, NAL for non-amputated leg and CG for control group.

**Table 1.** Description of the databases consulted, key words and combinations used, results achieved and types of experimental delineation among the papers found

Databases	Key words	Results	Types
Cochrane Library	gait and amputation	27	23 clinical trials
			3 systematic reviews
			1 economic evaluation
PubMed	locomotion and gait and biomechanics and amputee	74	14 clinical trials
			1 randomized controlled trial
			1 systematic review
			4 reviews
			45 observational cross-sectional studies
			1 observational cohort study
			6 case reports
			1 repeatability study
			1 validation study
SciELO	amputee	3	3 observational cross-sectional studies
Lilacs	gait and amputation	10	2 clinical trials
			7 observational cross-sectional studies
			1 observational prospective study
Dedalus	biomechanics and amputee and locomotion	1	1 observational cross-sectional study

## RESULTS

### Biomechanics of locomotion for transtibial amputees

Kinematics focuses on the measuring of gait among lower-limb amputees through recording variables such as speed, cadence, stride length and single stance duration. These were some of the first variables to be described in this population.^3^^,^^4^ Kinematics can be defined as the field of movement description, irrespective of the force caused. With regard to such parameters, we found different combinations of results between PL and NAL,^3^^,^^5^^,^^6^^,^^7^ marked by individual characteristics and by the components of the prostheses used in different studies.

Isakov et al.^5^ analyzed the natural speed of walking (1.25 m/s) among fourteen volunteers with prostheses composed of patellar tendon bearing (PTB) sockets and solid ankle cushion heel (SACH) feet. They found greater stride duration, swing duration and stride length in the PL, whilst the stance duration and single stance duration were shorter. The shorter single stance duration was consequent to the SACH foot, since its rigid ankle speeds up the weight transfer from the heel to the forefoot, thus resulting in shorter stance duration for the PL and the swing phase for the NAL.

Breakey^3^ found results similar to those of Isakov et al.^5^ regarding stance duration for the PL. Soares^6^ found different results from analysis of gait at a average speed (1.2 m/s) among five amputee athletes with dynamic response feet. The duration of the stride and stance were similar for the PL and the NAL, but the swing time was shorter in the PL.

Different results were found by Isakov et al.^7^ from analyzing the effect of speed on the symmetry between the limbs of fourteen amputees with SACH feet (nine with PTB sockets and five with a supracondylar tibial prosthesis) at a natural walking speed (0.9 m/s) and a fast speed (1.4 m/s). The stance duration, double-limb stance duration, step duration and step length were symmetrical at the two speeds and in the pairs of legs. Asymmetry was found for the angular variation of the knee. At the time of load response, as expected, there was increased flexing of the knee in the NAL on moving from the natural speed to the fast speed. This did not happen in the PL. Furthermore, at the time of foot release, there was greater flexing of the knee in the PL because of absence of dorsiflexion in the prosthetic foot.

Analysis of prosthetic feet while walking is commonly approached through such parameters, as done by Powers et al.^18^ in analyzing five feet (Carbon Copy II, Seattle, Quantum, SACH and Flex foot) among ten volunteers at a natural speed. The speed and cadence, irrespective of the foot, were similar between the legs (NAL and PL) and groups (amputees and CG), whereas the length of the Flex foot stride (1.5 m) was greater than the length of the SACH (1.44 m) and Quantum (1.44 m), while all the others were similar (Carbon Copy II 1.46 m, Seattle 1.47 m and CG 1.51 m). Regarding the joint trajectories, only the ankle presented differences. The Flex foot achieved greater dorsiflexion (23.2º) than the others: Quantum 19.5º, Carbon Copy II 12.1º, Seattle 15.1º and SACH 12.0º, and the Quantum dorsiflexion was greater than that of the SACH, Seattle and Carbon Copy feet.

Through kinematic parameters, it is also possible to analyze the progress of rehabilitation among lower-limb amputees. Baker and Hewison^8^ used speed analysis as a performance index. They demonstrated increased speed as a result of rehabilitation. It must be stressed that the increase was more notable over the first fifteen days following discharge of the initial weight.

To improve the understanding of inter-limb symmetry, Dingwell et al.^9^ used a combination of kinematic and kinetic parameter analysis. Kinetics is the field of biomechanics that analyses internal and external forces. Internal forces come from muscle or ligament activity, or from the friction between the muscles and joints. External forces in human locomotion come from the GRF that acts on the human body and throughout the locomotor system. Thus, through using a monitored treadmill, the pressure centre, stance duration and the second peak of the vertical GRF component were recorded. In comparisons between PL and NAL, the variables of stance duration and second peak of the vertical GRF component were asymmetrical. To reduce this asymmetry, training was carried out with visual feedback from the variables analyzed, and the single stance duration was taken as the variable test. The training reduced the asymmetry in the three variables trained, but the variable test either improved or worsened. This result shows that reducing the asymmetry in one variable does not result in improvement of others involved in the same phenomenon.

Prior use of GRF to analyze inter-limb symmetry is an important indicator of mechanical overload imposed on the lower limbs.^6^^,^^12^^,^^13^^,^^14^ Chronic abnormalities of gait, as occur in cases of lower-limb amputation, may lead to degenerative problems such as meniscus lesions.^22^

Through analysis of the variables relating to the first peak of the vertical GRF component, several studies have found that the mechanical shock in the PL of unilateral transtibial amputees when walking is lower than in the NAL and lower than among non-amputees.^6^^,^^12^^,^^13^^,^^14^^,^^17^

Soares^6^ and Tonon and Avila^14^ reported smaller magnitude and slower growth in the vertical GRF component in the PL. Lewallen et al.^23^ found the same in children, which was related to an attempt to increase the inter-limb symmetry. According to these authors, the smaller magnitude and slower growth of the force was a consequence from reductions in the speed of gait and length of stride, and increases in the duration of double limb stance and support in the PL, compared with the NAL.

Soares^6^ analyzed the gait of five amputee athletes and found similar results for the growth of the vertical component at natural walking speed (1.2 m/s), comparing PL and NAL. Unlike the abovementioned authors, this author stated that the symmetry was possibly related to the use of flexible feet and the high level of functional capacity in the sample. In the same study, an increase in speed (1.6 m/s) again generated smaller magnitude and slower growth in the vertical component.

Such asymmetry suggests that the NAL experiences higher overload than the PL does. To study this hypothesis, Hurley et al.^24^ analyzed the internal forces of joint reactions in the hips, knees and ankles of unilateral transtibial amputees, and found similar results for the hips, in comparisons between PL and NAL. For the knee and ankle, throughout the stance phase and especially in the first 25% of the cycle, the horizontal reaction forces were greater in the NAL. This differed from the CG, which presented inter-limb symmetry.

Barth et al.^13^ came up with results that indicated the dependence of the force on the prosthetic foot. In this case, at self-selected speeds, the Carbon Copy II and Quantum feet presented higher force values during the load response in the PL, while the SACH, SAFE, Seattle and Flex feet resulted in symmetry between the supports.

Menard et al.^12^ analyzed different kinds of feet (Flex and Seattle) and identified differences in comparisons between the CG and NAL. In the NAL with the Flex foot, the first peak of the vertical component (Fy1) was lower and the time taken to achieve it was greater than seen in the CG, whereas in the Seattle foot, Fy1 was greater, with a similar time interval. With regard to the mediolateral and anteroposterior components, the PL presented symmetry. In this case, the mediolateral component presented greater intensity in the PL, whereas the anteroposterior component showed lower values for peaks and impulses for propulsion and braking.

Mizuno et al.^15^ analyzed the GRF associated with spatial-temporal parameters to characterize 12 different prosthetic feet (three uniaxial, two multiaxial and seven rigid feet). Symmetry indices were calculated: 1- stride length rate; 2- speed rate; 3- efficiency of deceleration and acceleration; 4- magnitude of deflexing of the vertical GRF component from the first to the second peak; and 5- patterns presented by the anteroposterior component of the GRF. This was done from the rates in the PL and NAL to compare them with the rates from the left and right feet of the CG. Greater symmetry was found among the amputees for all variables. In addition to the influence of the prosthetic foot, these variables were influenced by the length of the stump, the level of muscle force from the lower limbs and the time interval since the amputation.

Studies on GRF among amputee populations have focused on analysis of the vertical component,^6^^,^^9^^,^^12^^,^^14^^,^^15^^,^^17^ while few have explored the anteroposterior^12^^,^^15^ and mediolateral^12^ components. Zmitrewicz et al.^25^ drew attention towards analysis of the anteroposterior component, because this leads to understanding of the specific parameters relating to the braking and propulsion phases. These authors analyzed the influence of the number of axes and the type of foot on the anteroposterior component and spatial-temporal parameters. With regard to axes, a multiaxial system was incorporated in the SACH and Carbon Copy II feet. Insertion of the axis did not influence the speed and cadence developed. The same was found for the stance duration, step length, braking impulse, ratio between the peaks and force peaks in the braking phase, and the propulsion of both supports. However, the NAL propulsion impulse increased after insertion of the axis in both feet. It can be noted that the number of axes in the prosthetic foot may have an influence on the behavior of the NAL.

Through the force platforms used in GRF analysis, it is also possible to analyze the trajectory of the centre of pressure (COP) and to understand the postural adjustments made during specific gait events, such as gait initiation^26^ and gait termination^27^ and the gait initiation process when stepping up and stepping down to a new level.^28^ The strategies used for gait initiation when the PL is used are marked by reduction or even absence of transference from the COP posteriorly, in conjunction with reduction in the first force peak for all three GRF components.^26^ However, in gait termination, there is a reduction in transference from the COP anteriorly and a reduction in the braking force. Thus, the trajectory of the COP increases mediolaterally.^27^ Furthermore, through kinematics, it has been shown that the PL is used preferentially for starting movement and the NAL for stopping it, while in both tasks, there are greater propulsive forces and braking in the NAL.^26^^,^^27^ In an earlier study,^29^ the same authors pointed out that to overcome an obstacle during the gait trajectory, unilateral transtibial amputees used the PL preferentially. Thus, increased knee flexion was the main strategy used in the control group to overcome the obstacle, and this was found to be similar among the amputees studied.^29^

Jones et al.^28^ stated that, to start the gait process when stepping up and stepping down to a new level, amputees preferentially used the PL for stepping up and the NAL for stepping down. In these cases, in beginning the process with the PL, the COP was directed anteriorly, unlike in the control group, in which it was directed posteriorly. This behavior reduced the extensor moment generated in the knee joint of the PL and increased the speed of movement during stepping up, in relation to stepping down. This strategy differed from that presented by the control group, for which the speed was greater when stepping down, since the posterior trajectory of the COP was greater. In this way, the amputees minimized the instability generated during stepping down consequent to the slope of the terrain and reduced the extensor moment required by the knee of the PL.

To improve the understanding of the strategies developed during walking, consequent to amputation, the moments of mechanical power and joint work need to be analyzed. Winter and Sienko^2^ analyzed the moment and power of the hip, knee and ankle joints in the sagittal plane among eight amputees (five SACH, two uniaxial and one Greissinger foot) during walking at a natural speed. They found that absence of the sural triceps had a direct influence on the adaptation of the joints near to the amputation level. Thus, the PL presented an initial dorsiflexor moment that was three times greater than in the CG, because of the delayed contact of the SACH and Greissinger feet with the ground. Since these feet were only deformed slightly, the energy absorbed and returned in the ankle region was small, and therefore the flexor plantar moment generated during propulsion by the PL was about 60-70% of that seen in the CG. During propulsion in the Greissinger foot, 30% of the energy absorbed was returned. In the uniaxial foot, the return was 20%, and in the SACH foot, energy was dissipated through its rigid material. In the knee, the moment and mechanical power were almost zero during the load response and midstance. Before propulsion, the flexor moment and negative power were found to be the same as in the CG, but during the swing phase, these values were near to zero. The absence of plantar flexors increased the energy absorption in the hip at the beginning of the stance and consequently increased the power generated at the end.

To determine whether dynamic response feet (Flex and Seattle feet) behave in a similar way to rigid feet (SACH), Gitter et al.^10^ investigated not only the joint moments and power but also the variation in energy indicated by the work done by the joints in the lower limbs of unilateral transtibial amputees. In the ankles of the CG, two power phases were found: the first related to energy absorption up to midstance (9.96 J) and the second related to generation during propulsion (26.42 J). The amputees presented a first phase that was similar to that of the CG (7.22, 9.40 and 18.24 J for the SACH, Seattle and Flex feet, respectively). However, the second phase was slow and significantly smaller (2.80, 6.71 and 16.20 J). The first and last power phases in the hip joint were significantly greater among the amputees. In the CG, the hips presented a positive phase (5.51 J) at the beginning of the stance, followed by a negative phase (13.67 J) and, at the end of the stance, a positive phase (2.93). This triphasic pattern was similar among the amputees, i.e. positive (10.73, 7.34 and 13.63 J for the SACH, Seattle and Flex feet, respectively), negative (4.51, 4.56 and 5.39 J) and positive (5.35, 4.00 and 5.01 J). The knee joint was shown to be capable of energy absorption in the CG (8.49 J). This was not found among the amputees (SACH 0.39 J, Seattle 1.59 J and Flex 2.13 J), who presented greater energy absorption in the hips.

In the same study, the authors also calculated an index to represent mechanical efficiency, from the results relating to energy absorbed and generated by each foot. This variable was defined as the proportion of energy generated during release of the foot that came from the energy absorbed during the first half of the stance. The proportion for the SACH foot was 39%, Seattle foot 71%, Flex foot 89% and CG 264%. Prince et al.^20^ had aims similar to those of Gitter et al.^10^ in analyzing the mechanical efficiency of different prosthetic feet. In their study, the Golden-Ankle foot achieved the best results (40.9%), followed by the Seattle (38.7%) and SACH (36.6%). In analyzing the results from these two studies, it can be noted that there is a discrepancy in the results from the Seattle foot, and this deserves further study.

With regard to the behavior of the knee joint moment, Sanderson and Tokuno^11^ found that one of the factors responsible for values of practically zero related to abnormalities of muscle activation. These authors found that the knee extensor moment in the PL was 50% smaller than that seen in the NAL, from analysis on amputees’ walk. In the same study, through EMG, the correlation found between the vastus lateralis muscle and semitendinosus muscles in the PL was less than the correlation in the CG.

EMG records the electrical energy associated with muscle contraction stemming from the power of the motor units.^30^ One of the first studies that analyzed the electromyographic activity relating to the locomotion of amputees was done by Winter and Sienko.^2^ The muscles studied in three amputees (SACH feet) presented different adaptations. The hip extensors, gluteus maximus, biceps femoris and semitendinosus muscles were dominant during most of the stance phase, in the same way as the vastus lateralis and rectus femoris muscles. Thus, there was coactivation of the hamstrings and the quadriceps during the first 40% of the stride. After this period, the hamstring activity diminished, but the quadriceps remained active until the end of the stance.

Focusing on the muscles that control the knee joint, Isakov et al.^5^ analyzed scores relating to the ratio between the biceps femoris and vastus medialis muscles for the intervals from 0 to 50% and 50 to 100% of the stance and swing phases of amputees’ walk. The prostheses used were the PTB socket and SACH foot. The score for the PL (3.8) over the 0 to 50% stance interval was significantly greater than that of the NAL (2.0). This was because the activity of the biceps femoris muscle in the PL (26.85) during the first half of the stance was twice as great as in the NAL (15.19). The values for the vastus medialis in the PL and NAL (8.45 and 9.75, respectively) were similar. For the remaining time intervals, the scores were similar. According to these authors, the increased activation of the biceps femoris stabilizes the knee joint during the initial instants of the stance.

Isakov et al.^16^ described new results from EMG on the vastus medialis and biceps femoris muscles of amputees with prostheses similar to those described previously. In this study, peak muscle action was assessed. In the vastus medialis in the NAL, this occurred after 6.06% of the walking cycle (WC), which was earlier than in the PL (8.84% of the WC) but without any significant difference. In the biceps femoris muscle of the NAL, the peak was at the end of the swing (92.43% of the WC), unlike in the PL, in which it occurred at the beginning of the stance (9.81 ± 4.8% of the WC). This difference related to the lower muscle force found in the remaining muscles. In other words, for adequate control over the lower limb during load response, the biceps femoris muscle was activated vigorously in conjunction with the quadriceps at the beginning of the stance phase.

Soares et al.^17^ found different results regarding the peak action of the biceps femoris muscle, in analyzing the walk of three amputee athletes at natural walking speed (1.5 m/s) and fast walking speed (1.8 m/s). The peak of the stride in the PL occurred at 97% and 98% of the WC, and in the NAL at 91% and 90% of the WC, at the end of the swing phase. The physical capacity of these athletes may have led to these results. In the same study, it was found that at the beginning of the stance phase, the vastus lateralis muscle in the PL remained active for up to 40% of the WC, whereas in the NAL, its action was until reaching 30%. The biceps femoris muscle followed the same tendency, i.e. in the PL, its action occurred until reaching 20% of the WC and in the NAL until reaching 5%. It can be noted that, in both cases, there is greater coactivation of these muscles in the PL, as pointed out by Winter and Sienko.^2^

Analysis of electromyographic activity is also a tool used to investigate the effect of different prosthetic feet on locomotion. Culham et al.^31^ analyzed EMG profiles from the vastus lateralis muscle and hamstrings of amputees with prostheses composed of PTB sockets and two different feet: SACH and uniaxial. For each foot, the subjects were given a period for adaptation, and the interval between data collections was 24 days. The foot used influenced the hamstrings. With the SACH foot, the peak muscle activity occurred at 30% of the WC, whereas for the uniaxial foot, two peaks were observed: the first at 10% of the WC and the second at the transition from stance to swing.

Torburn et al.^19^ used EMG associated with kinematic parameters to analyze different prosthetic feet (SACH, Flex, Carbon Copy II, Seattle and Sten). The vastus lateralis, gluteus maximus and biceps femoris muscles were analyzed by means of wire electrodes. No differences were found in comparing the muscle linear envelope with different kinds of feet. However, the EMG results from this study were greater than those described for normal subjects.^32^ With regard to angular variation, the Flex foot presented greater dorsiflexion in the preswing than the others did. Upon asking the subjects for their preferences, they chose the feet with dynamic response: the model most cited was the Seattle, which differed from the other regarding speed.

To investigate the strategies used by elderly subjects who had undergone amputation for vascular reasons, during climbing and descending ramps using the SACH foot, Vickers et al.^33^ analyzed the electromyographic activity of the vastus lateralis, lateral hamstring and gluteus maximus muscles in conjunction with spatial-temporal parameters, GRF and kinetic and kinematic variables. All the results were compared with a CG of similar age. In the PL, the muscles analyzed (gluteus maximus, vastus lateralis and lateral hamstring) presented greater magnitude of EMG activity and, during periods of activation, factors that contributed directly towards reducing the speed of movement and the vertical component of the GRF. In conjunction, there were reductions in cadence, stride length, angular variation of the knee and hip and moment in the hip joint in the PL. This study drew attention to the behavior of the contralateral leg because the reduction in this leg was found to be in the unilateral stance phase, along with reductions in the moment and power of all three lower-limb joints. These results for the contralateral, non-amputated leg demonstrate that walking on an inclined surface increases the demands on the locomotor system among this population. This is directly related to the absence of distal muscles, particularly the sural triceps, and the absence of mobility in the prosthetic ankle.

To minimize the asymmetries while walking in situations that require greater skill, focusing on going down stairs, and to optimize the control over the impact and the generation of power, Au et al.^34^ developed a motorized prosthetic foot controlled by sensorial impulses from the muscles in the stump in conjunction with data picked up by external sensors. The external sensors measured the ankle joint position, the contact of the heel and toes with the ground and the joint torque. Through the myoelectrical signals, amputees were able to control the movement of the prosthetic foot. In other words, when they wanted to walk on a flat surface, they activated the anterior tibial muscle, thereby causing dorsiflexion in the prosthetic foot. To go down stairs, they activated the plantar flexors, from which the prosthetic ankle performed plantar flexion to begin the descent. In initial evaluations, these authors found that the foot they developed was capable of simulating human movement, through generating energy during the propulsion phase of the stride and increasing the shock absorption in going down stairs.

## DISCUSSION

The complexity of human locomotion is approached in this population through combined analysis of different parameters in each of the fields of biomechanics.^35^^,^^36^^,^^37^^,^^38^^,^^39^^,^^40^^,^^41^^,^^42^ Only in this way is it possible to extrapolate from subjective analysis of the movement, to reach an understanding of the factors that could be of use for rehabilitation of amputees.

At this level of amputation, the absence of plantar flexors can be considered to be the main factor negatively influencing locomotion. The increased absorption and generation of energy in the hip joint of the PL can be considered to be the main compensatory strategy developed by unilateral transtibial amputees during gait. This conclusion can be reached through analysis of the joint moment, power and work, with confirmation through EMG. Thus, the hip joint and the muscles that control it need to receive special attention during the rehabilitation of transtibial amputees.

With regard to the biomechanical tools used for analyzing human movement, it needs to be made clear that the results relating to joint moment, power and work, as well as the EMG, explain the findings described by the kinematic parameters and the GRF.

From analysis on spatial-temporal parameters, it is seen that the sample characteristics and the prosthetic components analyzed have an influence on the results presented in various studies. Rigid feet lead to a fast step from foot strike to toe off, which causes changes in the behavior of the PL during the stance phase and in the NAL during the swing phase. Dynamic feet produce different behavior, with increased symmetry between the PL and NAL during the stance and swing phases. This relates to the elasticity of these feet, which gives rise to a more harmonic transition between foot strike and toe-off during the stance phase, since they provide greater range of motion for the prosthetic ankle.

With regard to the GRF, it can be noted that the behavior of the vertical component is linked to the smaller magnitude and slower growth at the beginning of the stance phase. It can be seen that dynamic response feet may make the behavior of the PL similar to the NAL. Smaller magnitude and slower growth of the vertical component point towards mechanical overload in the PL, and the GRF is lower in the initial instants of stance. Moreover, it has been shown that greater speed of movement is not accompanied by higher GRF values, especially with regard to the variables relating to the acceleration phase of the center of mass during propulsion.

The trajectory of the COP indicates that the postural adjustments made during the different phases (gait initiation and termination) and specific tasks (stepping up and stepping down to a new level) minimize body oscillation in the anteroposterior direction. This strategy compounds the reduced external force and joint angle changes, thereby increasing the balance control in tasks that require greater skill to perform. Hence, such activities must be included in the rehabilitation process for this population.

With regard to electromyographic activity, it can be noted that walking gives rise to increases in the number and duration of muscle action pulses. This leads to an increase in co-contraction moments between agonistic and antagonistic muscles. Co-contraction of the quadriceps and hamstrings, for example, explains the joint moment in the knee, which is practically zero at the beginning of the stance phase. Periods of coactivation may occur because of attempts to prevent the collapse of the lower limb during the initial instants of stance. Some muscles responsible for such a task, such as the tibialis anterior and the extensor digitorum longus, are absent at this level of amputation and therefore coactivation develops in order to increase joint rigidity.

Irrespective of the analytical tool used, it has been noted that the different characteristics of amputation and prostheses, and the different experimental protocols adopted hinder the production of a synthesis of this subject from the literature. Hence, strict inclusion and exclusion criteria were not established, as these might have restricted the knowledge that could be gained regarding this subject. The present text has thus been significantly influenced by these characteristics.

Among the experimental protocols, there has been notable variation in the speed of movement taken as the natural speed. Gait speeds over the ground ranging from 0.9 m/s to 1.4 m/s^7^ and 1.5 m/s^6^ on the treadmill has been used in different analyses. This indicates that if a standard speed for analyses on this population were to be imposed, erroneous interpretations of the movement could arise.

With regard to the prosthesis, special interest in analysis on the influence of the prosthetic foot on the movement has been shown in the literature. In a recent meta-analysis,^35^ it was sought to develop suitable criteria for prescribing prosthetic feet, based on functional characteristics. From a survey of the literature, 23 articles were selected. The meta-analysis was performed by analyzing the following parameters: speed, stride length, cadence, energy cost, step efficiency and Borg scale. These authors pointed out that there was no evidence to support surveying of specific criteria for prosthesis prescription. In addition, the Flex foot was shown to be superior to the SACH foot in only one study that analyzed ascending and descending gait. This indicates that although several studies in the literature describe the influence of prosthetic components on gait among this population, new studies need to be carried out with proper methodological control, so that in the future it will be possible to determine objective criteria for prosthesis prescription. Moreover, such studies may also be of help in issues relating to the rehabilitation process for amputees.

With regard to inter-limb symmetry, Dingwell et al.^9^ presented an important discussion on this subject. There have been several studies on symmetry as a measurement index for the efficiency of walk among amputees.^7^^,^^8^ According to Winter and Sienko,^2^ structural asymmetry in amputees creates adaptations to the musculoskeletal and nervous systems that consequently lead to an asymmetric pattern. These authors therefore rejected the presumption that efficiency of walk among unilateral transtibial amputees is linked to symmetry.

As the understanding of locomotion among these individuals improves through knowledge of their biomechanical parameters, amputees’ subjective perceptions regarding their movement could perhaps be better explored through concomitant studies. It may then be possible to arrive at a single conclusion regarding the importance of symmetry in locomotion among this population. This concern needs to be given due regard in rehabilitation and restoration of locomotion for this population, since it has been raised through analysis of the literature.

## CONCLUSION

The present analysis showed that the strategy developed by unilateral transtibial amputees during gait is influenced by the speed of movement, laterality and prosthetic foot.

The space-time parameters and angular variation present asymmetry between the limbs. The differences relate mainly to the duration of the stride, stance and balance, the stride length and the angular variation of the knee and ankle. Sample characteristics such as the physical activity level in the experimental protocol, the speed of movement and the prosthetic components (such as the foot) have a direct impact on movement and can increase or decrease the asymmetries.

Attenuation of the GRF occurs in the PL, and this is seen mainly through slower growth and smaller magnitude of the vertical component and lower braking and propulsion impulses in the anteroposterior component.

Reductions in the flexor plantar moment and in the mechanical force of the ankle joint in the PL are direct results from the absence of the sural triceps. Dynamic response feet optimize the energy return during propulsion, but the values achieved are still below those seen among non-amputees.

With regard to joint moment, one characteristic is of interest in the knee joint: values that are practically zero at the beginning of the stance phase.

The absence of plantar flexors increases the energy absorption in the hip at the beginning of the stance and consequently increases the power generated at the end of this phase.

Electromyography enables comprehension of various aspects of the other variables analyzed. In relation to the physical activity of the muscles, there is an increase in quadriceps activity in conjunction with hamstring activity during the initial instants of the stance phase, which leads to a prolonged period of co-contraction. The descriptions of the behavior of the femoral biceps in the PL in relation to the intensity of activation and the peak moment of the action are marked by different findings.

Based on the studies presented, it can be noted that the analyses on walking among transtibial amputees present consistent results that make it possible to understand the strategies developed during this movement. However, the influence of the different characteristics of the amputation, prosthesis and experimental protocols creates a need for new studies that consider all the specific features relating to amputees’ situations.
